# Cytotoxicity, mode of action and antibacterial activities of selected Saudi Arabian medicinal plants

**DOI:** 10.1186/1472-6882-13-354

**Published:** 2013-12-12

**Authors:** Victor Kuete, Benjamin Wiench, Mansour S Alsaid, Muhammad A Alyahya, Aimé G Fankam, Abdelaaty A Shahat, Thomas Efferth

**Affiliations:** 1 Department of Pharmaceutical Biology, Institute of Pharmacy and Biochemistry, University of Mainz, Staudinger Weg 5, 55128 Mainz, Germany; 2 Department of Biochemistry, Faculty of Science, University of Dschang, P.O. Box 67, Dschang, Cameroon; 3 Pharmcognosy Department, College of Pharmacy, King Saud University, P.O. Box 2457, Riyadh 11451, Saudi Arabia; 4 Phytochemistry Department, National Research Centre, 12311 Dokki, Cairo, Egypt

**Keywords:** Antibacterial, Cytotoxicity, Mode of action, Medicinal plants, Saudi Arabia

## Abstract

**Background:**

The flora of Saudi Arabia is one of the richest biodiversity areas in the Arabian Peninsula and comprises very important genetic resources of crop and medicinal plants. The present study was designed to investigate the cytotoxicity and the antibacterial activities of the organic extracts from twenty six Saudi Arabian medicinal plants. The study was also extended to the investigation of the effects of the extracts from the four best plants, *Ononis serrata* (SY160), *Haplophyllum tuberculatum* (SY177), *Pulicaria crispa* (SY179), and *Achillea beiberstenii* (SY-200) on cell cycle distribution, apoptosis, caspases activities and mitochondrial function in leukemia CCRF-CEM cell line.

**Methods:**

A resazurin assay was used to assess the cytotoxicity of the extracts on a panel of human cancer cell lines whilst the microbroth dilution was used to determine the minimal inhibitory concentration (MIC) of the samples against twelve bacterial strains belonging to four species, *Escherichia coli*, *Enterobacter aerogenes*, *Klebsiella pneumoniae* and *Pseudomonas aeruginosa*.

**Results:**

The best activity on leukemia cell lines were recorded with SY177 (IC_50_ of 9.94 μg/mL) and SY179 (IC_50_ of 1.81 μg/mL) against CCRF-CEM as well as Ach-b (IC_50_ of 9.30 μg/mL) and SY160 (IC_50_ of 5.06 μg/mL) against HL60 cells. The extracts from SY177 and SY179 were also toxic against the seven solid cancer cell lines studied with the highest IC_50_ values of 31.64 μg/mL (SY177 against Hep-G2 cells). SY177 and Ach-b induced cell cycle arrest in G0/G1 and S phases whilst SY160 and SY179 induced arrest in G0/G1 phase. All the four plant extracts induced apoptosis in CCRF-CEM cells with the alteration of the mitochondrial membrane potential. In the antibacterial assays, only Ach-b displayed moderate antibacterial activities against *E. coli* and *E. aerogenes* ATCC strains (MIC of 256 μg/mL), AG100A_TeT_ and *K. pneumoniae* ATCC strains (MIC of 128 μg/mL).

**Conclusions:**

Finally, the results of the present investigation provided supportive data for the possible use of some Saudi Arabian plants investigated herein, and mostly *Haplophyllum tuberculatum, Pulicaria crispa*, *Ononis serrata* and *Achillea beiberstenii* in the control of cancer diseases.

## Background

Medicinal plants constitute an important source of new candidates for therapeutic compounds, in regards to the chemical diversity found in several species. Many plant-derived compounds are currently successfully employed in cancer chemotherapy, explaining the endeavor of researchers worldwide for the intensive search of new anti-neoplastic agents from the nature. The flora of Saudi Arabia is one of the richest biodiversity areas in the Arabian Peninsula and comprises very important genetic resources of crop and medicinal plants [1]. It was estimated that the flora of Saudi Arabia has a great medicinal species diversity, which is expected to be more than 1200 (over 50%) out of its 2250 species [1]. Hence, the present study was designed to investigate the cytotoxicity and the antimicrobial activities of twenty six Saudi Arabian plants against leukemia and carcinoma cell lines. In addition, the effects of the four best plants namely *Haplophyllum tuberculatum*, *Pulicaria crispa*, *Ononis serrata* and *Achillea beiberstenii* on cell cycle distribution, apoptosis, caspases activities and mitochondrial function in leukemia CCRF-CEM cells were investigated.

## Methods

### Plant material

All the plants were collected from Tanhat protected area except *Cleome ambliocarpa, Artemisia monosperma, Ononis serrata* and *Achillea beibersteni* that were collected from Um-Rugum, Aldahua, Kaba and Albaha, respectively, (Saudi Arabia) in March 2010. The plants were identified by the Plants Taxonomy and Herbarium Unit. The voucher specimens (Table 1) have been deposited at the Herbarium of the Faculty of Pharmacy, King Saud University, Riyadh, Saudi Arabia.

**Table 1:** Medicinal plants used in the present study

**Plant species/Family (Voucher specimen)**	**Traditional use**	Sample used* (yield in %)	**Index**
*Teucrium oliverianum/*Lamiaceae (15930)	Antinociceptive effect [2], antioxidant activity [3] and antimicrobial activities [4]	20.0	SY-175
*Echium arabicum/*Boraginaceae (15931)	Antiplasmodial and antitrypanosomal activity [5]	14.5	SY-176
*Haplophyllum tuberculatum/*Rutaceae (15932)	Headaches and arthritis, to remove warts and freckles from the skin and also to treat skin discoloration, infections and parasitic diseases [6], it is used to treat malaria, rheumatoid arthritis and gynecological disorders [7]	19.6	SY-177
*Senna italica*/Caealpiniaceae (15933)	Diarrhea, stomachache, female infertility, tuberculosis, asthma [8]	14.9	SY-178
*Pulicaria crispa*/Asteraceae (15934)	Treat inflammation and an insect repellent [9] and is also used as an herbal tea	6.7	SY-179
*Rhantarium epapposum/*Asteraceae (15935)	Skin infections and gastrointestinal disturbances and as an insecticide [10]	3.1	SY-180
*Rumex vasicanus/*Polygonaceae (15936)	Treatment of pain, inflammation, bleeding, tinea, tumor, and constipation [11,12], cough, headache, and fever [13]	13	SY-181
*Ducrosia anethifolia*/Aplaceae (15937)	Analgesic and pain reliever for headache, ackache, colic, and colds [14]	26	SY-182
*Heliotropium ramosissimum/*Boraginaceae (15938)	Treatment of gout, rheumatism, and as anti-inflammatory and healing agents [15]	2.58	SY-183
*Picris cyanocarpa/*Asteraceae (15939)	Treatment of indigestion, against intestinal nematodes and other parasites [16]	19.5	SY-184
*Anthemis deserti/*Asteraceae (15940)	Herbal medicines, insecticides, and dyes, food additives, as well as an important source in aromatic and cosmetic industries [17]	10.8	SY-185
*Cleome ambliocarpa/*Cleomaceae (15945)	Stomachics, rubefacients and in the treatment of scabies, rheumatic, fever, inflammation and a hypoglycemic agent, [18]	14.9	SY-186
*Zilla spinosa/*Brassicaceae (15946)	Antioxidant, antifungal, hepatoprotective and antiviral activities [19]	13.5	SY-187
*Ziziphus nummularia/*Rhamnaceae (15947)	Antibacterial, analgesic activities and anthelmintics [20]	11.6	SY-188
*Neurada procumbens/*Neuradaceae (15949)	Diarrhea and dysentery; as well, it has been used as a tonic to ‘increase heart and respiration functions	8.6	SY-189
*Trigonella hamosa/*Papilionaceae (15951)	A condiment and seasoning in food preparations and hypoglycemic	22	SY-190
*Achillia fragrantissima/*Asteraceae (15952)	Respiratory diseases and gastrointestinal disturbances [21]	6.2	SY-191
*Convolvulus prostates/*Convolvulaceae (15953)	Brain related disease, improve memory, skin disease [22]	15.3	SY-192
*Cltrullus colocynthis/*Cucurbitaceae (15954)	Treat constipation, diabetes, edema, fever, jaundice, bacterial infections as well as cancer [23]	14.2	SY-193
*Emex spinosa/*Polygonaceae (15955)	Purgative, diuretic, a remedy for stomach disorders, dyspepsia and colic [24]	16.8	SY-194
*Rhazya strict/*Apocynaceae (15957)	Diabetes mellitus, fever, sore throat, inflammatory conditions and helminthiasis [25]	22	SY-195
*Scrophularia hypericifolia/*Scrophulariaceae (15958)	Antipyretic, febrifuge and anti-bacterial, as a remedy for evening fever, mouth dryness, constipation, prurigo, furunculosis, sore throat, ulcerous stomatitis, tonsillitis and in the treatment of cancer [26,27]	12.12	SY-196
*Caylusea hexagyna/*Resedaceae (15959)	Anticancer (melanoma cell lines) [28]	13.04	SY-197
*Artemisia monosperma/*Asteraceae (15960)	Antispasmodic, anthelmintic and anti-hypertensive [29]	16.4	SY-198
*Ononis serrata/*Fabaceae (15925)	Antibiotic, antipyretic, anti-inflammatory, antifungal and antiseptic activities. Treatment of skin and rheumatic diseases as well as gout [30]	5.5	SY160
*Achillea beiberstenii/*Asteraceae (15470)	Spasmolytic, choleretic, treatment of wounds and anti-inflammatory activities, make it as an important medicinal plant [31]	10.4	Ach-b

*80% methanol extracts from the aerial part.

### Extraction

The aerial parts of plants were collected and dried under shade. The dried samples were powdered and used for solvent extraction. For extract preparation, 100 g of dried sample was extracted with 80% methanol for 48 h and the procedure was repeated once. The extracts were filtrated through Whatman No. 1 and combined, then concentrated using a rotary evaporator under reduced pressure at 40°C. The dry extract obtained with each solvent was weighed. The percentage yield was expressed in terms of air dried weight of plant materials.

### Chemicals

Doxorubicin, vinblastine and daunorubicin from Sigma-Aldrich (Munich, Germany) were dissolved in Phosphate Buffer Saline (PBS; Invitrogen, Eggenstein, Germany) at a concentration of 10 mM. Geneticin was purchased from Sigma-Aldrich at a concentration of 50 mg/mL in sterile-filtered water. Chloramphenicol (Sigma-Aldrich, St Quentin Fallavier, France) was used as reference antibacterial drug.

### Cell cultures

Leukemia CCRF-CEM and HL-60 cells were maintained in RPMI 1640 medium (Invitrogen) supplemented with 10% fetal calf serum in a humidified 5% CO_2_ atmosphere at 37°C. Cells were kindly provided by Dr. J. Beck (Department of Pediatrics, University of Greifswald, Greifswald, Germany). Breast cancer cells transduced with control vector (MDA-MB-231-pcDNA3) or with cDNA for the breast cancer resistance protein *BCRP* (MDA-MB-231-*BCRP* clone 23) were maintained under standard conditions as described above for CCRF-CEM and HL-60 cells. Human wild-type HCT116 (*p53*^+/+^) colon cancer cells as well as knockout clones HCT116 (*p53*^-/-^) derived by homologous recombination were a generous gift from Dr. B. Vogelstein and H. Hermeking (Howard Hughes Medical Institute, Baltimore, MD). Non-transduced human glioblastoma multiforme U87MG cells and U87MG cells transduced with an expression vector harboring an epidermal growth factor receptor (*EGFR*) gene with a genomic deletion of exons 2 through 7 (U87MG.Δ*EGFR*) were kindly provided by Dr. W. K. Cavenee (Ludwig Institute for Cancer Research, San Diego, CA). MDA-MB-231-*BCRP,* U87MG.Δ*EGFR* and HCT116 *(p53*^-/-^*)* were maintained in DMEM medium containing 10% FBS (Invitrogen) and 1% penicillin (100 U/mL)-streptomycin (100 μg/mL) (Invitrogen) and were continuously treated with 800 ng/mL and 400 μg/mL geneticin, respectively. The multidrug resistance profile of these cell lines has been reported [32]. Human HepG2 hepatocellular carcinoma cells and the AML 12 normal heptocytes were obtained from the American Type Cell Culture Collection ATCC (USA). DMEM medium without geneticin was used to maintain MDA-MB-231, U87MG, HCT116 (*p53*^+/+^), HepG2 and AML 12 cell lines. The cells were passaged twice weekly. All experiments were performed with cells in the logarithmic growth phase.

### Resazurin reduction assay

Resazurin reduction assay [33] was performed to assess the cytotoxicity of the crude extracts towards various sensitive and resistant cancer cell lines. The assay is based on reduction of the indicator dye, resazurin, to the highly fluorescent resorufin by viable cells. Non-viable cells rapidly lose their metabolic capacity to reduce resazurin and, thus, do not produce fluorescent signals anymore. Briefly, adherent cells were detached by treatment with 0.25% trypsin/EDTA (Invitrogen) and an aliquot of 1 × 10^4^ cells was placed in each well of a 96-well cell culture plate (Thermo Scientific, Germany) in a total volume of 200 μL. Cells were allowed to attach overnight and then were treated with different concentrations of compounds. For suspension cells, aliquots of 2 × 10^4^ cells per well were seeded in 96-well-plates in a total volume of 100 μL. The studied sample was immediately added in varying concentrations in an additional 100 μL of culture medium to obtain a total volume of 200 μL/well. After 24 h or 48 h, 20 μL resazurin (Sigma-Aldrich, Germany) 0.01% w/v in double distilled water (ddH_2_O) was added to each well and the plates were incubated at 37°C for 4 h. Fluorescence was measured on an Infinite M2000 Pro™ plate reader (Tecan, Germany) using an excitation wavelength of 544 nm and an emission wavelength of 590 nm. Each assay was done at least two times, with six replicate each. The twenty six studied plants were first tested on CCRF-CEM and HL60 at 40 μg/mL and samples inducing less than 50% growth proliferation were further diluted serially (in a concentration ranges of 0.33 to 40 μg/mL) and tested for IC_50_ determinations. Afterwards, samples with IC_50_ value below and around 30 μg/mL on the above leukemia cells were further tested on the solid cancer cells in a concentration ranges of 0.33 to 40 μg/mL. The viability was evaluated based on a comparison with untreated cells. IC_50_ values represent the compound’s concentrations required to inhibit 50% of cell proliferation and were calculated from a calibration curve by linear regression using Microsoft Excel.

### Determination of cell cycle distribution and apoptosis by flow cytometry

The cell cycle analysis was performed by flow cytometry using the Vybrant® DyeCycle™ Violet (Invitrogen). This dye is a DNA-selective, cell membrane-permeant, and non-fluorescent stain that uses the violet laser for DNA content analysis in living cells. Vybrant® DyeCycle™ Violet is fluorescent upon binding to double-stranded DNA. Leukemia CCRF-CEM cells (1 × 10^6^) were treated with concentrations equivalent to the IC_50_ values of the four most active plant extracts namely those from *Ononis serrata* (SY160), *Haplophyllum tuberculatum* (SY177), *Pulicaria crispa* (SY179), and *Achillea beiberstenii* (SY-200), for 24 h. Following incubation, 1 μL of Vybrant® DyeCycle™ Violet was added to 1 mL of cell suspension and incubated for 30 min at 37°C. Cells were measured on a LSR-Fortessa FACS analyzer (Becton-Dickinson, Germany). For each sample 10^4^ cells were counted. Vybrant® DyeCycle™ Violet was measured at an excitation of 440 nm. Cytographs were analyzed using FlowJo software (Celeza, Switzerland).

### Caspase-Glo 3/7, caspase-Glo 8 and caspase-Glo 9 assay

The influence of extracts SY160, SY177, SY179, and Ach-b on caspase 3/7, caspase 8 and caspase 9 activities in CCRF-CEM leukemia cells was detected using Caspase-Glo 3/7, Caspase-Glo 8 and Caspase-Glo 9 Assay kits (Promega, Germany). Cells cultured in RPMI were seeded in 96-well plates and treated with the sample (2 × IC_50_ and IC_50_) or DMSO (solvent control). After 6 h treatment, 100 μL of caspase reagent were added to each well, mixed and incubated for 1 h at room temperature. Luminescence was measured using well Infinite M2000 Pro™ instrument (Tecan). Caspase activity was expressed as percentage of the untreated control.

### Analysis of mitochondrial membrane potential (MMP)

The effects of extracts SY160, SY177, SY179, and Ach-b on the MMP were analyzed by 5,5′,6,6′-tetrachloro-1,1′,3,3′-tetraethylbenzimidazolylcarbocyanine iodide) (JC-1; Biomol, Germany) staining. JC-1 is a dye that can selectively enter into mitochondria and exhibits an intense red and green fluorescence in healthy mitochondria with normal membrane potentials. In cells with reduced MMP, the red fluorescence disappears and the unhealthy cells show only green fluorescence. Briefly, 1 × 10^6^ CCRF-CEM cells treated with different concentrations of compound or DMSO (solvent control) for 24 h were incubated with JC-1 staining solution according to the manufacturer’s protocol for 30 min. Subsequently, cells were measured in a LSR-Fortessa FACS analyzer (Becton-Dickinson). For each sample, 1 × 10^4^ cells were counted. The red JC-1 signal was measured with 561 nm excitation (150 mW) and detected using a 586/15 nm bandpass filter. The green JC-1 signal was analyzed with 488 nm excitation (25 mW) and detected using a 530/30 nm bandpass filter. All parameters were plotted on a logarithmic scale. Cytographs were analyzed using FlowJo software (Celeza, Switzerland).

### Antibacterial assay

#### Bacterial strains and culture media

The studied microorganisms included the reference (from the American Type Culture Collection) and multidrug-resistant (MDR) clinical strains of *Escherichia coli* (ATCC8739 and AG100ATET), *Enterobacter aerogenes* (ATCC13048 and CM64), *Klebsiella pneumoniae* (ATCC11296 and KP63) and *Pseudomonas aeruginosa* (PA01 and PA124). They were maintained in Nutrient Broth supplemented at 4°C and activated on a fresh appropriate Mueller Hinton Agar plates 24 h prior to any antimicrobial test. The Mueller Hinton Broth (MHB) was also used for the all antibacterial assays.

#### Bacterial susceptibility determinations

The MICs were determined using the rapid INT colorimetric assay [34,35]. Briefly, the test samples were first dissolved in DMSO/MHB. The solution obtained was then added to MHB, and serially diluted two fold (in a 96- wells microplate). One hundred microlitres (100 μL) of inoculum (1.5 × 10^6^ CFU/mL ) prepared in MHB was then added. The plates were covered with a sterile plate sealer, then agitated to mix the contents of the wells using a shaker and incubated at 37°C for 18 h. The final concentration of DMSO was 2.5% and does not affect the microbial growth. Wells containing MHB, 100 μL of inoculum and DMSO at a final concentration of 2.5% served as negative control (this internal control was systematically added). Chloramphenicol was used as reference antibiotic. The MICs of samples were detected after 18 h incubation at 37°C, following addition of 40 μL of a 0.2 mg/mL INT solution and incubation at 37°C for 30 minutes. Viable bacteria reduce the yellow dye to pink. MIC was defined as the lowest sample concentration that exhibited complete inhibition of microbial growth and then prevented this change [36]. All assays were performed in triplicate and repeated thrice.

#### Statistical analysis

Statistical analysis of all data was performed using a Student’s *t*-test or Kruskal–Wallis test followed by Dunn’s post-hoc multiple comparison test (Graph-Pad Prism 5.01; GraphPad Software, Inc., CA, USA). A significance level of *P* < 0.05 denoted significance in all cases.

## Results

### Cytotoxicity

**Figure 1:**
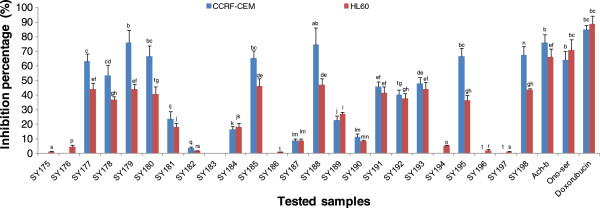
**Inhibitory percentage (%) of plant extracts at 40 μg/mL and doxorubicin (10 μg/mL) on leukemia CCRF-CEM and HL60 cancer cell lines.** Data with different superscript letters are significantly different (P < 0.05).

In the present work, the cytotoxicity of twenty six Saudi Arabian plants (Table 1) was initially evaluated against leukemia CCRF-CEM and HL60 cell lines. The results depicted in Figure 1 show that only the extract from *Heliotropium ramosissimum* (SY183) did not prevent the growth of the two cell line. Besides, those from *Teucrium oliverianum* (SY175), *Echium arabicum* (SY176) and *Emex spinosa* (SY194) did not prevented the growth of CCRF-CEM cells. All other extracts were able to inhibit in various extents the proliferation of CCRF-CEM as well as HL60 cells. More than 50% inhibition of the growth of CCRF-CEM and HL60 were exhibited by the extracts from *Achillea beiberstenii* (SY-200; 76.27% and 66.22% respectively) as well as *Ononis serrata* (SY160; 64.32% and 71.16% respectively). Also, more than 50% growth inhibition of CCRF-CEM was recorded with *Haplophyllum tuberculatum* (SY177; 63.21%), *Senna italica* (SY178; 53.57%), *Pulicaria crispa* (SY179; 76.17%), *Rhantarium epapposum* (SY180; 66.71%), *Anthemis deserti* (SY185; 65.54%), *Ziziphus nummularia* (SY188; 74.99%), *Rhazya strict* (SY195; 66.63%) and *Artemisia monosperma* (SY198; 67.66%). The IC_50_ values of the ten above samples were then determined on CCRF-CEM cells whilst those of Ach-b and SY160 were evaluated on HL60 cells (Table 2). Apart from SY178 (IC_50_ value of 37.13 μg/mL) and SY180 (IC_50_ value of 37.13 μg/mL) against CCRF-CEM cells, other tested extracts displayed values below or around 30 μg/mL. The best activities were recorded with SY177 (IC_50_ of 9.94 μg/mL) and SY179 (IC_50_ of 1.81 μg/mL) against CCRF-CEM as well as Ach-b (IC_50_ of 9.30 μg/mL) and SY160 (IC_50_ of 5.06 μg/mL) against HL60 cells. Samples with IC_50_ value below and around 30 μg/mL (SY177, SY179, SY185, SY188, SY189, SY198, SY160 and Sch-b) were further tested on the solid cancer cells (Table 3) including both sensitive and resistant phenotypes. The results showed that only the extracts from SY177 and SY179 were toxic to the seven solid cancer cells with the highest IC_50_ values of 31.64 μg/mL (SY177 against Hep-G2 cells). Other samples showed selective activities, the extracts of SY160 and Ach-b being active on six of the seven solid cancer cells tested. Consequently, the extracts SY160, SY177, SY179, and Ach-b which showed the best activities were further investigated for their effects on the cell cycle distribution, apoptosis, caspase 3/7, 8 and 9 activities as well as on MMP using CCRF-CEM cells as model for the studies.

**Table 2:** **IC**_**50**_**values of Saudi Arabian plants towards leukemia cancer cell lines CCRF-CEM and HL60 as determined by the resazurin assay**

**Tested samples**	**Samples code**	**Cell lines and IC**_**50 **_**(μg/mL)**	
		**CCRF-CEM**	**HL60**
*Haplophyllum tuberculatum*	SY177	9.94	-
*Senna italica*	SY178	37.13	-
*Pulicaria crispa*	SY179	1.81	-
*Rhantarium epapposum*	SY180	31.9	-
*Anthemis deserti*	SY185	30.08	-
*Ziziphus nummularia*	SY188	16.6	-
*Rhazya strict*	SY195	22.35	-
*Artemisia monosperma*	SY198	19.64	-
*Ononis serrata*	SY160	17.44	9.30
*Achillea beiberstenii*	SY-200	18.11	5.06
Doxorubicin		0.11	0.40

(-): >40 μg/mL.

**Table 3:** Cytotoxicity of Saudi Arabian plants towards sensitive and drug-resistant solid cancer cell lines and normal cells as determined by the resazurin assay

**Tested samples**	**Sample code**	**Cell lines, IC**_**50**_(μg/mL ) and degree of resistance*							
		**MDA-MB-231/pcDNA**	**MDA-MB-231/BCRP**	**HCT116** ***p53***^**+/+**^	**HCT116** ***p53***^**-/-**^	**U87MG**	**U87MG** ***ΔEGFR***	**HepG2**	**AML 12**
*Haplophyllum tuberculatum*	SY177	11.92	26.65 (2.24)	2.42	3.30 (1.36)	15.03	27.67 (1.84)	31.64	-
*Pulicaria crispa*	SY179	14.6	8.37 (0.57)	2.79	2.58 (0.92)	4.31	27.46 (6.37)	27.85	-
*Anthemis deserti*	SY185	-	-	24.48	31.94 (1.30)	-	-	-	-
*Ziziphus nummularia*	SY188	-	-	-	-	-	-	-	-
*Neurada procumbens*	SY189	-	-	-	-	3.01	-	-	-
*Artemisia monosperma*	SY198	-	26.16 (<0.65)	7.35	6.65 (0.90)	25.25	24.95 (0.99)	-	-
*Ononis serrata*	SY160	19.28	25.09 (1.30)	14.9	21.94 (1.47)	28.58	31.09 (1.09)	-	-
*Achillea beiberstenii*	SY-200	10.48	22.32 (2.13)	17.09	17.31 (1.01)	-	19.24 (<0.48)	27.28	-
Doxorubicin		1.10	7.83 (7.12)	1.41	4.06 (2.88)	1.06	6.11 (5.76)	1.41	-

(*): The degree of resistance was determined as the ratio of IC_50_ value of the resistant/IC_50_ sensitive cell line; (-): >40 μg/mL.

### Cell cycle distribution and apoptosis

**Figure 2:**
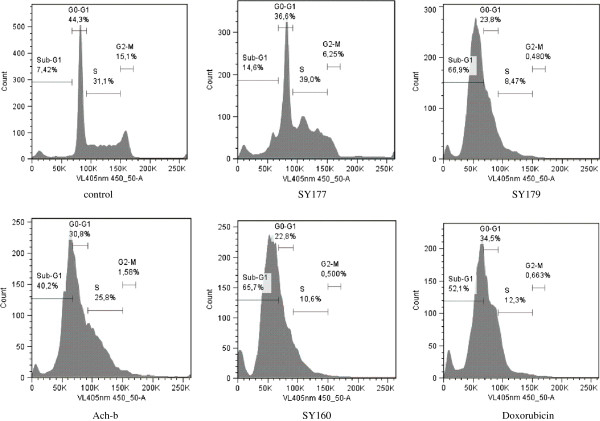
**Cell cycle distribution of leukemia CCRF-CEM cells treated with selected plant extracts and doxorubicin at their IC**_**50 **_**values for 24 h.**

The effects of extracts SY160, SY177, SY179, and Ach-b on the cell cycle distribution of CCRF-CEM cells are summarized in Figure 2. All the four extracts as well as doxorubicin considerably altered the distribution of the different cell cycle phases after 24 h. All the four extracts also significantly induced apoptosis after 24 h treatment with percentages of sub-G0/G1 phase of 14.6% for SY177, 66.9% for SY179, 40.2% for Ach-b and 65.7% for SY160.

### Effect on the mitochondrial membrane potential (MMP)

**Figure 3:**
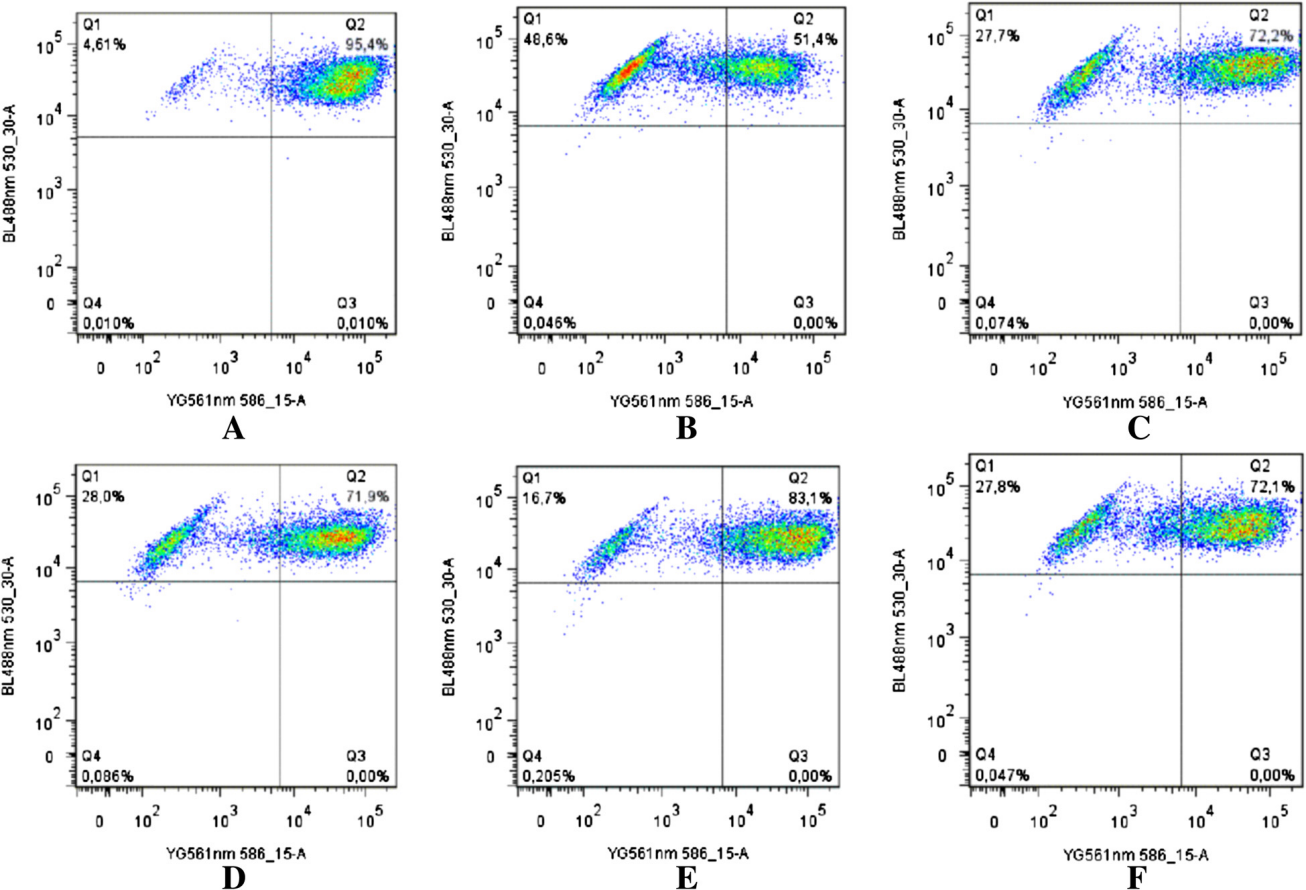
**Effect of selected plant extracts and vinblastine on the MMP of CCRF-CEM cells after 24 h treatment at their IC**_**50 **_**values; The IC**_**50 **_**values: 0.20 μM for VIN (this study).** Samples: control **(A)**, vinblastine **(B)**, SY177 **(C)**, SY179 **(D)**, Ach-b **(E)**, SY160 **(F)**. Q1 represent cells with healthy MMP whilst Q2-Q4 are those with altered MMP.

Since the breakdown of the MMP is amongst the sequences of events occurring during the apoptotic pathway, we assessed the involvement of extracts SY160, SY177, SY179, and Ach-b as well as that of the reference drug vinblastine in the process. The results summarized in Figure 3 show that the four extracts as well as vinblastine were able to alter the MMP in CCRF-CEM upon 24 h treatment. When cells were treated with concentrations equivalent to the IC_50_ values of the samples, percentages of alterations of the MMP observed were found to be 16.7%, 27.7%, 27.8% and 28.0% respectively for Ach-b, SY160, SY177, and SY179. However, such alteration was still lower than that of vinblastine (48.6%).

### Effect on the activity of caspases 3/7, 8 and 9

The influence of extracts SY160, SY177, SY179, and Ach-b on caspases 3/7, 8 and 9 activities in CCRF-CEM cells were investigated. None of the four samples induced the activation of each of the studied enzymes (data not shown).

### Antimicrobial activity

The twenty six plant extracts tested for their cytotoxicity were also, screened for their antibacterial activities against eight bacterial strains belonging to four species, *Escherichia coli*, *Enterobacter aerogenes*, *Klebsiella pneumoniae* and *Pseudomonas aeruginosa*. Only Ach-b displayed moderate antibacterial activities against *E. coli* and *E. aerogenes* ATCC strains (MIC of 256 μg/mL), AG100A_TeT_ and *K. pneumoniae* ATCC strains (MIC of 128 μg/mL). This extracts as well as the twenty-five others were not active on bacterial species.

## Discussion

According to the criteria of the American National Cancer Institute, as reported by Suffness and Pezzuto [37], 30 μg/mL is the upper IC_50_ limit considered promising for purification of a crude extract. Therefore, the highest concentration tested (40 μg/mL) in our screening was slightly above this limit. Herein, IC_50_ values below 30 μg/mL were recorded with the crude extract from SY160 and Ach-b against the two leukemia cell lines, CCRF-CEM and HL60 (Table 2). In addition, the two plant extracts also displayed cytotoxic activities with IC_50_ values below 30 μg/mL against six of the seven solid cancer cells (Table 3). This highlights the role of these two plants as potential source of products to combat both hematological and solid cancers. Also the extracts from SY177 and SY179 showed good activities against the leukemia CCRF-CEM cells (IC_50_ value of 9.94 and 1.81 μg/mL respectively) and all the seven studied solid cancer cells, with the IC_50_ values below 10 μg/mL against MDA-MB231*-BCRP* (8.37 μg/mL for SY179), HCT116 WT (2.42 μg/mL for SY177 and 2.79 μg/mL for SY179). HCT116 *(p53-/-)* (3.30 μg/mL for SY177 and 2.58 μg/mL for SY179) and against U78MG (2.58 μg/mL for SY179). These data also suggest that the two plant extracts can be potential source of cytotoxic agents. This is remarkable, since the extracts also exhibited considerable activity towards genetically modified cell lines, which harbor cDNAs encoding for proteins mediating resistance to standard anticancer drugs, such as the breast cancer resistance protein (BCRP) and an mutation-activated epidermal growth factor receptor (EGFR) or which were knocked-out in the expression of the tumor suppressor p53. Importantly, the degrees of resistance of the four most active plant extracts *Ononis serrata* (SY160), *Haplophyllum tuberculatum* (SY177), *Pulicaria crispa* (SY179), and *Achillea beiberstenii* (SY-200) were generally lower than that of doxorubicin for the corresponding drug-resistant cell lines (Table 3). Besides, collateral sensitivity (resistant cells being more sensitive than their sensitive parental cell lines with degrees of resistance below 1) was observed with each of the four plant extracts on at least one MDR cell lines (Table 3). This suggests that the extracts from *Haplophyllum tuberculatum*, *Pulicaria crispa*, *Ononis serrata* and *Achillea beiberstenii* might be useful to fight MDR cancer cells. Interestingly, normal AML12 hepatocytes were more resistant to the extracts than HepG2 hepatocellular carcinoma cells. This may be taken as a hint that these compounds exhibit some tumor specificity, although their toxicity towards normal organs needs to be further explored. Consequently, the four best plant extracts, namely those from *Ononis serrata* (SY160), *Haplophyllum tuberculatum* (SY177), *Pulicaria crispa* (SY179), and *Achillea beiberstenii* (SY-200) were selected for further mechanistic studies. It was observed that SY160, SY177, SY179, and Ach-b induce apoptosis in leukemia CCRF-CEM cells (Figure 2). The percentages of sub-G0/G1 phase upon treatment with SY160 (65.7%) and SY179 (66.9%) were higher than those of doxorubicin (52.1%) (Figure 2) highlighting their strong apoptotic inducing effects. SY177 and Ach-b induce cell cycle arrest in G0/G1 and S phases whilst SY160 and SY179 induce arrest in G0/G1 phase (Figure 2). Caspases, or cysteine-aspartic proteases are a family of cysteine proteases that play essential roles in apoptosis, necrosis, and inflammation [38]. As the extracts SY160, SY177, SY179, and Ach-b were found to strongly induce apoptosis in CCRF-CEM cells (Figure 2), we evaluated the involvement of initiator caspases 8 and 9 as well as that of effector caspases 3 and 7 in this process. Unfortunately, none of the four studied extracts (SY160, SY177, SY179, and Ach-b) activated these enzymes (data not shown), suggesting that it is not the main pathways involved in apoptosis in CCRF-CEM cells. Hopefully, when assaying their effects on MMP, the four plant extracts were found to induce the dysfunction of the mitochondrial membrane in CCRF-CEM cells (Figure 3). The alteration of the MMP can therefore be suggested as one of the possible mechanisms of apoptosis induced by SY160, SY 177, SY179, and Ach-b.

Phytochemicals are routinely classified as antimicrobials on the basis of susceptibility tests that produce MIC in the range of 100 to 1000 mg/mL [39]. Activity is considered to be significant if MIC values are below 100 μg/mL for crude extracts and moderate when 100 < MIC < 625 μg/mL [40]. In this study, only Ach-b displayed moderate antibacterial activities against *E. coli* and *E. aerogenes* and *K. pneumoniae*. Therefore, none of the studied samples could be considered as promising source of antimicrobial compounds.

## Conclusions

Finally, the results of the present investigation highlight the cytotoxic potential of the studied Saudi Arabian medicinal plant extracts, especially those from *Haplophyllum tuberculatum*, *Pulicaria crispa*, *Ononis serrata* and *Achillea beiberstenii*. The isolation of active constituents from these plants will further be performed.

## Competing interests

The authors declare that they have no competing interest.

## Authors’ contributions

VK*,* BW, STL, MSA and AGF carried out the study; VK, AAS and TE designed the experiments. VK and AAS wrote the manuscript; VK and TE supervised the work; all authors read and approved the final manuscript.

## Pre-publication history

The pre-publication history for this paper can be accessed here:

http://www.biomedcentral.com/1472-6882/13/354/prepub
